# Understanding the Interaction Modes and Reactivity of Trimedoxime toward *Mm*AChE Inhibited by Nerve Agents: Theoretical and Experimental Aspects

**DOI:** 10.3390/ijms21186510

**Published:** 2020-09-05

**Authors:** Alexandre A. de Castro, Daniel A. Polisel, Bruna T. L. Pereira, Elaine F. F. da Cunha, Kamil Kuca, Eugenie Nepovimova, Teodorico C. Ramalho

**Affiliations:** 1 Department of Chemistry, Federal University of Lavras, 37200-000 Lavras, Brazil; alexandre.a.castro@hotmail.com (A.A.d.C.); dpolisel@yahoo.com.br (D.A.P.); bru.limapereira92@gmail.com (B.T.L.P.); 2 Biomedical Research Center, University Hospital Hradec Kralove, 500 05 Hradec Kralove, Czech Republic; 3 Department of Chemistry, Faculty of Science, University of Hradec Kralove, 500 03 Hradec Kralove, Czech Republic; eugenie.nepovimova@uhk.cz

**Keywords:** nerve agents, acetylcholinesterase, trimedoxime, reactivation, mechanistic studies, computational methods

## Abstract

Organophosphorus (OP) compounds are used as both chemical weapons and pesticides. However, these agents are very dangerous and toxic to humans, animals, and the environment. Thus, investigations with reactivators have been deeply developed in order to design new antidotes with better efficiency, as well as a greater spectrum of action in the acetylcholinesterase (AChE) reactivation process. With that in mind, in this work, we investigated the behavior of trimedoxime toward the *Mus musculus* acetylcholinesterase (*Mm*AChE) inhibited by a range of nerve agents, such as chemical weapons. From experimental assays, reactivation percentages were obtained for the reactivation of different AChE–OP complexes. On the other hand, theoretical calculations were performed to assess the differences in interaction modes and the reactivity of trimedoxime within the AChE active site. Comparing theoretical and experimental data, it is possible to notice that the oxime, in most cases, showed better reactivation percentages at higher concentrations, with the best result for the reactivation of the AChE–VX adduct. From this work, it was revealed that the mechanistic process contributes most to the oxime efficiency than the interaction in the site. In this way, this study is important to better understand the reactivation process through trimedoxime, contributing to the proposal of novel antidotes.

## 1. Introduction

Chemical weapons are defined as any chemical substance whose toxic properties are used for the purpose of killing, injuring or incapacitating an enemy in war or associated military operations [1,2,3]. Even with the efforts from world entities to ban the use of chemical weapons, under the Chemical Weapons Convention, diverse countries still have an arsenal of these chemical substances [4,5,6]. Among these chemical weapons, the most toxic to humans are the well-known nerve agents, whose base structure consists of an organophosphorus compound (OP). In addition, a range of these toxic substances show potential for application in agricultural and industrial sectors [7].

From this class of OP substances, the pesticides are fundamental in agroindustrial applications [8]. Although the OP compounds are widely used for pest control, they are very dangerous and toxic to humans, animals, and the environment. These sorts of compounds act by inhibiting the acetylcholinesterase (AChE) enzyme causing a cholinergic neurotoxic effect. In the presence of OP, the Ser203 residue from the AChE active site covalently binds to the phosphorus atom forming a phosphorylated complex [2,9]. The AChE inhibition causes an accumulation of acetylcholine (ACh), once this enzyme is responsible for the hydrolysis of this neurotransmitter. This toxic framework results in ACh accumulation, giving rise to a cholinergic syndrome, which is a set of symptoms associated with poisoning from certain toxic substances, such as OP nerve agents, caused by the overstimulation of muscarinic and nicotinic receptors [10]. Among the major symptoms of the intoxication, we can cite excessive salivation, lacrimation, urination, sweating, broncho-constriction and neuromuscular block, leading to death in severe cases of poisoning [10,11,12].

**Figure 1:**
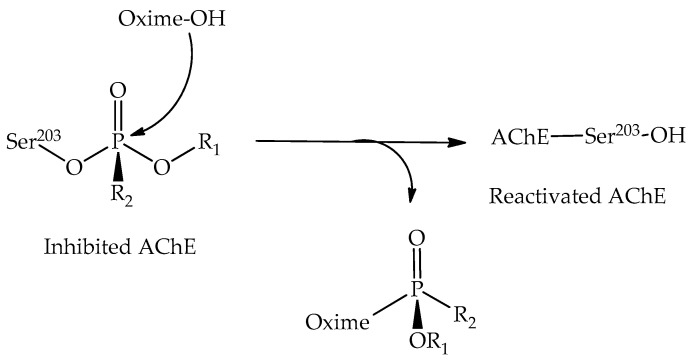
General representation of the reactivation process of the inhibited acetylcholinesterase (AChE).

The current treatment protocol for OP poisoning consists mainly of the employment of a reactivating agent, commonly an oxime compound [13,14,15,16], which is capable of restoring the AChE catalytic activity through a nucleophilic attack, thus remediating the intoxication effects and reestablishing the ACh levels [17,18,19]. The general reactivation mechanism through oximes is represented in Figure 1.

**Figure 2:**
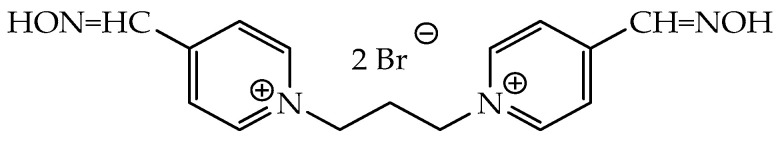
Chemical structure of trimedoxime.

In view of what has been exposed so far, it is important to note that there is no universal antidote to date, that is, a broad-spectrum oxime capable of reactivating all types of OP-inhibited AChE. In recent years, efforts have focused on the screening and identification of potent oximes, with sufficient permeability through the blood–brain barrier (BBB), maintaining a high reactivation rate [19,20,21]. In this study, we present a theoretical and experimental investigation to better understand the reactivation mechanism of the AChE inhibited by several kinds of OP agents. Based on interaction and mechanistic studies, we seek to explain the experimental data through molecular modeling in order to comprehend the reactivation process, by employing trimedoxime as the reactivating species (Figure 2). We expect to understand the interaction modes and reactivity of trimedoxime in the reactivation process of the AChE–OP adduct.

## 2. Results and Discussion

### 2.1. In Vitro Test: Experimental Results

**Figure 3:**
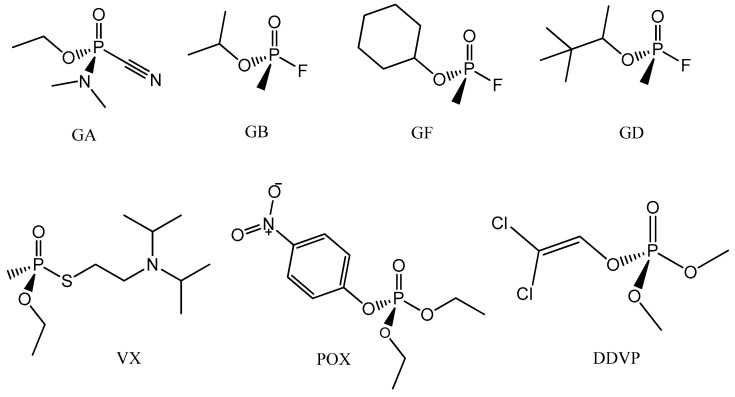
Chemical structures of the nerve agents used in the work.

The results obtained through the experimental part of this work are summarized in Table 1 and Figure 3. 

According to the literature, the efficiency (reactivation percentage) of an oxime should be at least 10% to provide an appropriate remediation for the intoxicated patient [22,23]. In this regard, we observe different reactivation percentages through trimedoxime, taking into account its concentration as well as the type of OP–AChE complex. According to our experimental findings, note that trimedoxime demonstrated the best results at higher concentrations (10^−3^ M). At this concentration, the oxime showed a remarkable reactivation percentage of 85.3% for AChE–VX reactivation. At a concentration of 10^−3^ M, trimedoxime also exhibits a good performance in the reactivation of the AChE–sarin (GB) (54%) and AChE–paraoxon (POX) (46%) adducts. This result was more modest for AChE–Tabun (GA) (30%). An interesting outcome from this experimental investigation is the fact that trimedoxime does not reactivate the AChE–cyclosarin (GF) and AChE–soman (GD) adducts. These trends are more deeply approached in the next sections. From the experimental assays with trimedoxime at lower concentrations, we can observe that the experimental values indicate a significant reactivation percentage for AChE–POX (50%), as well as a sufficient reactivation rate for AChE–Dichlorvos (DDVP) (17.3%). Indeed, this oxime showed insufficient reactivating power for the AChE inhibited by the other OP agents investigated, such as GA, GF and GD, considering a concentration of 10^−5^ M. The chemical structures of the nerve agents used in this work are shown in Figure 3. 

### 2.2. Affinity and Thermodynamics: Docking Results

According to the docking protocol, calculations were performed in order to investigate the affinity between trimedoxime and inhibited AChE. For this, a cavity prediction algorithm based on a 3D box was used to find the binding sites in the inhibited enzyme active site. The active cavity presented a volume of 113.66 Å^3^, which was appropriate to support the reactivator.

The molecular mechanics-based calculations generated diverse poses of trimedoxime within the cavity of the inhibited complexes, and the respective intermolecular interaction energy was computed to each system. As usual in these computations, the best oxime conformation was chosen for subsequent quantum mechanics (QMs) calculations, based on the lowest interaction energies as well as the most reactive conformations. Table 2 shows the values obtained from the docking calculations for the most appropriate doses of trimedoxime with different inhibited complexes.

According to the data reported in Table 2, note that trimedoxime showed stabilizing interactions within the inhibited enzyme complex site for all the OP agents investigated. From these results, the oxime demonstrated the lowest interaction energy in the AChE–DDVP (−164.8 kcal mol^−1^) adduct, followed by AChE–GF (−161.3 kcal mol^−1^) and AChE–GD (−157.7 kcal mol^−1^). In turn, the oxime showed a less stabilizing interaction energy within the AChE–VX cavity. As shown in the experimental section, at higher concentrations, the trimedoxime demonstrated a better efficiency in the reactivation of the AChE–VX adduct. This trend leads us to believe that the interaction energy is not the only factor responsible for the performance of this antidote in the reactivation, but other factors should be involved. In this regard, the results from the mechanistic study are presented in the next section.

From Table 2, the trimedoxime was stably docked in the inhibited AChE, with intermolecular interaction energy values in the range of −115.0 to −164.8 kcal mol^−1^. Diverse kinds of intermolecular interactions contribute to the stabilizing interaction on the site, such as hydrophobic interactions, electrostatic interactions and hydrogen bonds. It is important to mention that the AChE active site adopts distinct conformations according to the sort of OP agent. Thus, it is expected that trimedoxime interacts differently with residues from the active site. These hydrogen bond-type interactions are generally the most important in studies of biological systems.

**Figure 4:**
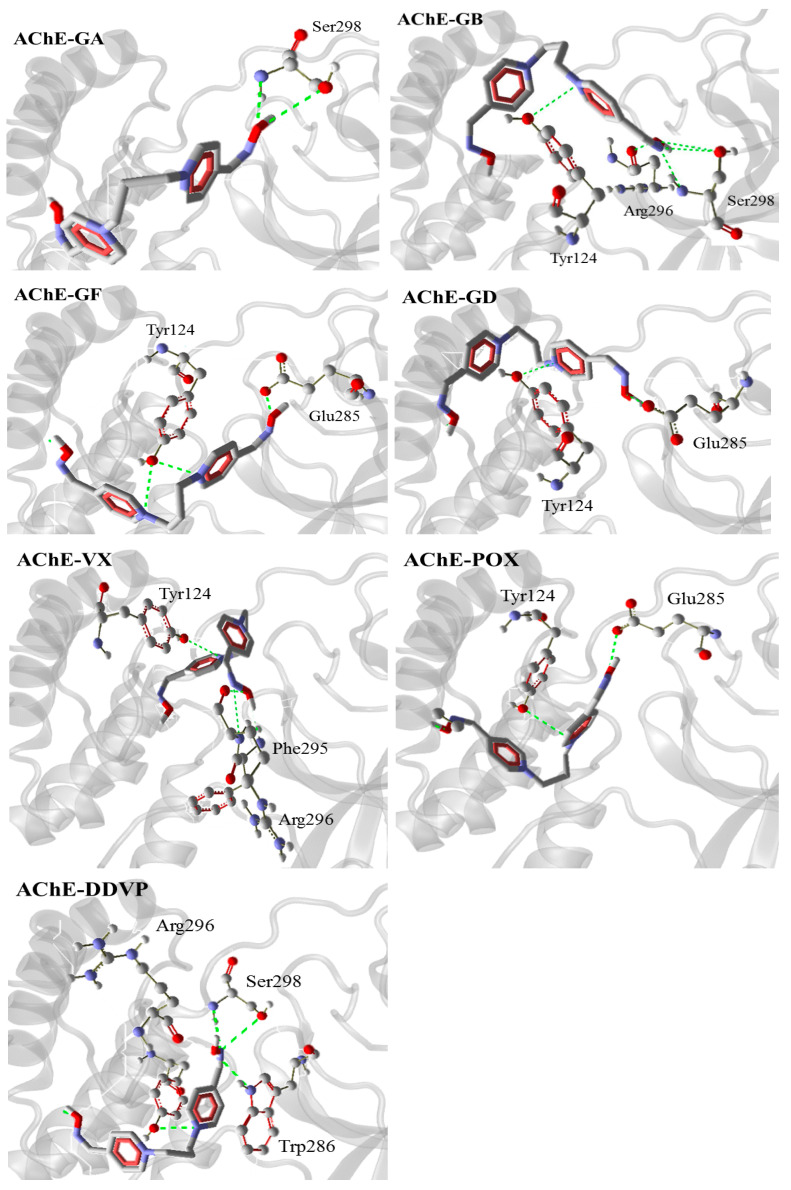
Representation of the hydrogen bonds performed by trimedoxime in the site.

In most of the systems investigated, trimedoxime interacted with the Tyr124 amino acid residue, and according to the literature, this interaction is described as a possible π–π stacking, which takes place between Tyr124 residue and the pyridine ring of the oxime. This interaction is indicated as having an important role in helping transition state stabilization [2,24,25]. The hydrogen bonds revealed by the interaction of trimedoxime in each inhibited system are shown in Figure 4.

From what has been discussed so far, it is important to notice that, together with the reactivation percentage, the interaction energy data do not explain the experimental trends thoroughly. The discussion of the mechanistic studies in the next section will give rise to new insights about the behavior of trimedoxime toward different AChE–OP systems.

### 2.3. Investigating Kinetic Parameters for Biological Activity: Mechanistic Studies

In the last part of this investigation, theoretical calculations were carried out to determine the relative activation energy (∆∆E^#^) through the hybrid QM/molecular mechanics (MMs) for the reactivation of each inhibited AChE system. The ∆E^#^ values were computed based on the energy difference between the transition states and the initial system configurations from the reactants.

For the reaction mechanism simulation, the steric and electronic effects of the chemical reactions are important aspects of the reaction pathway. In addition, the strain and interaction energies are significant contributing factors that dictate the reaction course. The interaction energy is responsible for stabilizing the reaction. On the other hand, the strain energy is responsible for distorting the reactants to adopt a pentacoordinate transition state. The relation between interaction and strain energies determines the height of the reaction barrier (∆E^#^), the so-called activation energy. This parameter was elucidated for some of these reactions in order to better comprehend trimedoxime’s behavior in the reactivation process. For this, a combined procedure of docking and DFT calculations at the QM/MM interface for the mechanism was carried out. The transition states were characterized through potential energy curves. Table 3 shows the kinetic parameters ∆∆E^#^, as well as the experimental values of reactivation at the concentration of 10^−3^ M.

According to Table 3, these quantum theoretical results corroborate our experimental findings. Trimedoxime has shown itself to be very efficient in reactivating the inhibited AChE–VX at a concentration of 10^−3^ M, which is according to the reactional barrier observed in the reactivation of this inhibited complex. From Table 3, the reactivation of the AChE–VX adduct revealed the lowest barrier. This fact helps explain the higher experimental reactivation percentage of the AChE inhibited by VX, which was 85.3%. This fact suggests that its transition state is better stabilized, allowing for the oxime to interact more strongly with the nerve agent.

As we can see from Table 3, the reactivation of the AChE–GB complex showed the second most stabilizing barrier (33.43 kcal mol^−1^), which corroborates the second best reactivation percentage found in our experimental assays (54%). In addition, a barrier of 41.59 kcal mol^−1^ was computed for AChE–POX, and for the AChE–GA and AChE–DDVP, our computations indicate very close barriers, 46.83 and 47.75 kcal mol^−1^, respectively, which correlate very well with the close experimental reactivation percentages found for these respective systems. It is worth mentioning that our simulations for the reactivation of the AChE–GF and AChE–GD did not succeed in our study, that is, the AChE inhibited by these OP agents did not provide a feasible conformation for the nucleophilic attack by trimedoxime on the active site. This fact could be explained by the interaction modes of these toxic agents on the site, mostly due to steric hindrance effects, as well as intermolecular interactions.

From the ΔΔE^#^ values in Table 3, we performed a multiple linear regression (MLR) between this parameter and the reactivation percentage, as well as the interaction energy. Our results revealed that the combination of interaction energy (ΔE) and activation energy (ΔΔE^#^) is able to efficiently explain the experimental outcomes. By increasing the number of system descriptors, a better correlation between theory and experiment is expected. Based on this, the MLR between the experimental and theoretical parameters resulted in the equation below. The regression was obtained with an excellent correlation value of 0.97.

% reactivation = −0.14Δ*E* − 1.22ΔΔ*E*^#^ + 70.85 (1)

By analyzing Equation (1), we can observe some important trends about the studied systems. Starting with the correlation value from the MLR, it shows that the docking conjugated to the QM/MM calculations result in a better representation of the systems investigated. According to the coefficients of the equation, the importance of each stage for the AChE reactivation process by trimedoxime can be evaluated. Note that the highest modulus of the coefficient of the term ΔΔ*E*^#^ (relative activation energy) indicates that the reaction step presents a greater contribution to the AChE reactivation than the interaction energy [23,25]. This means that trimedoxime can more easily fit to the transition state structure in the reactivation process. In addition, the binding mode of trimedoxime in the site is not a critical step for activity. With the information exposed in this investigation, we observe that trimedoxime stands for a significant advance in the development of more efficient reactivators for the remediation of the intoxication caused by neurotoxic nerve agents.

Previous studies have shown that there is not a direct correlation in oxime-mediated reactivation between species, and comparative studies in one species may not truly reflect the reactivation effects in humans. Due to structural differences, the active site of both enzymes from rats and humans may adopt distinct conformations in the presence of the neurotoxic agent, and the antidote might be led to specific reactional behaviors. In this context, in silico and in vitro investigations with the human AChE are equally important. These aspects will be considered in future investigations [26].

## 3. Materials and Methods

### 3.1. Experimental Details

Trimedoxime was prepared at the department of Toxicology in School of Military Health Sciences (Czech Republic), according to the synthesis route described earlier [27]. The purity of the reactivator was detected through the thin layer chromatography (TLC) and high-performance liquid chromatography (HPLC) techniques and NMR [28]. All compounds were obtained from the Brno Military Facility (95% purity and higher).

The animals employed in this experiment were handled under the supervision of the Ethics Committee of the School of Military Health Sciences in Hradec Kralove, Czech Republic. As a source of cholinesterases, a 10% rat brain homogenate (*w*/*v*) was used. The homogenate was prepared as described: ether-narcotized rats (*n* = 6) were killed by bleeding from a carotid artery. The brain was removed, washed with saline and homogenized using an Ultra-Turrax homogenizer in distilled water.

For the in vitro test, 0.5 mL of the brain homogenate was mixed with 20 μL of the isopropanol solution of the selected nerve agent and distilled water (0.5 mL). The mixture was incubated for 30 min at 25 °C to achieve a 95% inhibition of AChE. In total, 2.5 mL of sodium chloride (3 M) and distilled water were added to a volume of 23 mL. Finally, 2 mL of the substrate—ACh iodide (0.02 M)—was added. The enzyme activity (analyzed by a potentiometric titration of the decomposed ACh iodide) was measured at pH 7.6 and 25 °C on an autotitrator RTS 822 (Radiometer, Denmark). The same procedure was undertaken with the inhibited enzyme, as was a further treatment with a 10 min incubation with an aqueous solution of the reactivator (0.2 mL of 10^−3^ M), which replaced 0.2 mL of water. The activities of intact AChE (*a*_0_), inhibited AChE (*a_i_*) and reactivated AChE (*a_r_*) were deduced from the consumption of the NaOH solution (0.01 M) over time; NaOH reacted with the acetate released from the decomposed ACh iodide. The reactivation percentage (%) was calculated from the measured data according to the formula (Equation (2)):

(2) $$X=\left(1-\frac{a_{0}-a_{r}}{a_{0}-a_{i}}\right)\cdot 100\;\left[\%\right]$$

The entire method is described in detail in the work from Kuca and Cabal [29]. This same methodology was successfully employed in the work from Polisel et al. (2019) [23].

### 3.2. Docking Procedure

In the docking studies, the affinity of trimedoxime with the AChE inhibited by diverse OP agents was investigated. The oxime chemical structure was constructed and optimized at the DFT level, with the B3LYP density functional method and 6-31g(d,p) basis set, as implemented in the Gaussian 09 package [30]. The oxime was then docked inside the crystallographic structure of *Mus musculus* AChE (PDB code 3ZLU; resolution = 2.60 Å) [31] inhibited by GA (Tabun), GB (Sarin), GF (Cyclosarin), GD (Soman), VX, POX (Paraoxon) and DDVP (Dichlorvos), using the Molegro Virtual Docker program (Molegro Virtual Docker (MVD^®^)) [32], according to similar procedures employed previously [24,33,34]. From our calculation protocol, a radius of about 20 Å was considered, where the residues of the catalytic triad were kept flexible. Due to the nature of the docking methods, the calculations carried out generated approximately 50 poses (such as conformation and orientation) for each ligand studied. 

In the MVD program, the MolDock score algorithm method used as a scoring function is based on the piecewise linear potential, which is fundamentally a simplified potential whose parameters are in turn fitted to protein–ligand structures, binding data scoring functions and further extended in the Generic Evolutionary Method for molecular docking, including a new hydrogen bonding term as well as new charge schemes [32]. Along this line, the docking scoring function values, *E_score_*, are usually defined by Equation (3):

(3) $$E_{score}=E_{inter}+E_{intra}$$

where in:

(4) $$E_{inter}=\underset{i\varepsilon ligand}{\sum }\underset{j\varepsilon protein}{\sum }\left[E_{PLP}\left(r_{ij}\right)+332.0\;\frac{qiqj}{4r_{ij}^{2}}\right]$$

Note that the *E_PLP_* stands for ‘‘piecewise linear potential’’, which consists of the use of two different parameter sets: one for the approximation of the steric term (i.e., Van der Waals) among atoms, as well as the other to assess the potential for hydrogen bonding. As can be seen, the second term is, of course, related to the electrostatic interactions among overloaded atoms. Typically, it is a Coulomb potential with a dielectric constant dependent on the distance (which can be approximately described as *D*(*r*) = 4*r*). Hence, for this, the numerical value of 332.0 is responsible for the electrostatic energy unit to be given in kilocalories per molecule, as well [32].

*E_intra_* is defined as the internal energy of each ligand. That is:

(5) $$E_{intra}=\underset{i\varepsilon ligand}{\sum }\underset{j\varepsilon ligand}{\sum }E_{PLP}\left(r_{ij}\right)+\underset{flexiblebonds}{\sum }A\left[1-\cos\left(m.\theta \;-\;\theta_{0}\right)\right]\;+\;E_{clash}$$

Note that the first part of the equation (double summation) is among all pairs of atoms in the ligand, taking off those connected by two bonds. Thus, in this equation, the second term denotes the torsional energy, where θ is the torsional angle of the bond. Hence, if several torsions could be determined, then each torsional energy value is considered as an average among them. The last term, *E_clash_*, assigns a penalty of about 1.000 if the distance between two heavy atoms (e.g., more than two bonds apart) is smaller than 2.0 Å, but does not take into account infeasible ligand conformations [32]. Thus, the docking search algorithm that is applied in the MVD program considers an evolutionary algorithm that is based on interactive optimization techniques (inspired by Darwinian evolution theory), which implies a new hybrid search algorithm conveniently called guided differential evolution. As such, this hybrid combines the differential evolution optimization technique with a cavity prediction algorithm during the search process, allowing a fast and accurate identification of potential binding modes (poses) [32,35,36].

### 3.3. QM/MM Procedure

In line with the large number of atoms present in the investigated systems, a quantum mechanics (QM)-based treatment becomes infeasible due to the high computational demand. However, the covalent bond rearrangements in the reactional process cannot be ignored and treated exclusively through molecular mechanics (MMs). In this context, hybrid quantum mechanics–molecular mechanics (QM/MM) were employed in this investigation in order to study the reaction pathway involved in the reactivation process [37]. From this protocol, the AChE active site was treated through QM methods, DFT in this case, and the rest of the system was treated with MM-based methods [38]. From these calculations, the energetic barrier of the reactivation process of each enzyme–OP complex with trimedoxime was determined. This theoretical strategy has been previously employed in other works [26,38,39,40,41,42,43]. The QM part of the calculations was performed through the Gaussian 09 package, at the DFT level and 6-31g(d,p) basis set [44,45]. The delimited QM region includes: Ser203 residue bound to the respective OP, the residues Tyr124, Phe295, Arg296, Glu285, Ser298 and Trp286, in addition to trimedoxime. In this simulation, all precursors, transition states and intermediates were calculated and characterized by identifying imaginary frequencies [25,46,47]. Each system was fully optimized at the DFT level with conjugate gradient and quasi-Newton–Raphson algorithms. The final geometries were obtained with the density functional Becke’s three-parameter exchange functional and the gradient-corrected functional of Lee, Yang and Paar (B3LYP) [35,48], by using a 6-31g(d,p) basis set.

## 4. Conclusions

In this work, we tested the in vitro efficiency of trimedoxime and applied computational techniques to evaluate the interaction modes and reactivity of this antidote in the reactivation process of the AChE inhibited by a range of OP nerve agents. Thus, the kinetic factors and interactions that govern the AChE enzyme reactivation process were investigated. With this in mind, our theoretical outcomes show that the active site of the inhibited AChE adopts different conformations according to the kind of neurotoxic agent. Therefore, these conformational changes in the site result in different interactions and a different reactivity of trimedoxime in the active cavity.

Our findings indicate that the performance of trimedoxime enhances by increasing its concentration; the best result found was for the reactivation of the AChE–VX adduct. On the other hand, our experimental results show that trimedoxime was inefficient in the reactivation of the AChE–GF and AChE–GD complexes. Interestingly, appropriate conformations were not found when simulating the reactivation mechanisms with these complexes, which can be explained, for instance, by the steric hindrance observed in the site, thus causing a significant conformational change in the cavity.

Through the MLR analysis, we can observe that the combination of interaction energy and reaction energy is sufficient to explain the experimental data with a high correlation. However, the mechanistic part has a greater weight and contributes most to the reactivation process through trimedoxime. Therefore, this work will bring about important contributions to the field of drug design and therapies, assisting in the development of a broad spectrum and more efficient reactivators.

## Figures and Tables

**Table 1:** Reactivation activity of trimedoxime (data obtained in triplicate experimental assays).

System	Trimedoxime	
	React. (%) Conc. 10^−5^ M	React. (%) Conc. 10^−3^ M
AChE-GA	0	30
AChE-GB	7	54
AChE-GF	0	0
AChE-GD	0	0
AChE-VX	9.8	85.3
AChE-POX	50	46
AChE-DDVP	17.3	31.5

**Table 2:** Docking results for trimedoxime inside different AChE–organophosphorus compound (OP) adducts.

System	Trimedoxime	
	∆E * (kcal mol^−1^)	Residues
AChE-GA	−140.9	Ser298
AChE-GB	−154.7	Tyr124, Ser298, Arg296
AChE-GF	−161.3	Tyr124, Glu285
AChE-GD	−157.7	Tyr124, Glu285
AChE-VX	−115.0	Tyr124, Phe295, Arg296
AChE-POX	−144.1	Tyr124, Glu285
AChE-DDVP	−164.8	Arg296, Ser298, Trp286

* ∆E = Intermolecular interaction energy.

**Table 3:** Experimental reactivation percentage and relative activation energy for trimedoxime in the reactivation process.

System	Trimedoxime	
	∆∆E^#^ * (kcal mol^−1^)	React. (%) Conc. 10^−3^ M
AChE-GA	46.83	30
AChE-GB	33.43	54
AChE-GF	-	0
AChE-GD	-	0
AChE-VX	0	85.3
AChE-POX	41.59	46
AChE-DDVP	47.75	31.5

* ∆∆E^#^ = Relative activation energy.

## Abbreviations

AChE	Acetylcholinesterase
*Mm*AChE	*Mus musculus* Acetylcholinesterase
OP	Organophosphorus compounds
ACh	Acetylcholine
BBB	Blood Brain Barrier
GA	Tabun
GB	Sarin
GF	Cyclosarin
GD	Soman
POX	Paraoxon
DDVP	Dichlorvos
SER	Serine
Tyr	Tyrosine
ARG	Arginine
GLU	Glutamic acid
PHE	Phenylalanine
TRP	Tryptophan
QM/MM	Quantum Mechanics/Molecular Mechanics
MLR	Multiple Linear Regression
TLC	Thin Layer Chromatography
HPLC	High-performance liquid chromatography
NMR	Nuclear Magnetic Resonance
MVD	Molegro Virtual Docker
